# Impacts of Coil Treatment on Anxiety and Depression in Emphysema

**DOI:** 10.1155/2020/4270826

**Published:** 2020-05-11

**Authors:** Tugce Toker Ugurlu, Erhan Ugurlu

**Affiliations:** ^1^Department of Psychiatry, Faculty of Medicine, Pamukkale University, Denizli, Turkey; ^2^Department of Pulmonology, Faculty of Medicine, Pamukkale University, Denizli, Turkey

## Abstract

Chronic obstructive pulmonary disease (COPD) is a widespread, preventable, and treatable disease. Emphysema is one of the primary components of COPD and manifests itself via decrease in elastic recoil, hyperinflation, and increase in air trapping. Various lung-volume-reduction treatments have come up in recent years for late-stage emphysema patients. Mental disorders and especially anxiety and depression are among the frequently encountered comorbid cases observed in COPD. The aim of our study was to examine the impact of coil treatment applied for late-stage COPD-emphysema diagnosed patients on the accompanying anxiety and depressive symptoms. A total of 21 patients diagnosed with emphysema that meet the suitability criteria for coil treatment were included in the study. The accompanying anxiety and depressive symptoms of the patients were assessed via beck anxiety inventory (BAI) and beck depression inventories (BDI-I) prior to the procedure and one month later. All patients were male with an age average of 66.5 ± 5.5 (57–76). Among patients without a psychiatric diagnosis, BAI scores before and after coil treatment were determined, respectively, as 12.1 ± 6.3 (4–26) and 11.2 ± 9.3 (0–28), whereas BDI-I scores before and after coil treatment were determined, respectively, as 13.5 ± 10.4 (1–31) and 8.8 ± 10.6 (0–34), with a statistically significant difference between them. Also among patients with a psychiatric diagnosis, both anxiety and depressive symptoms decreased after coil treatment, and this reduction was found more significant for anxiety. Coil treatment as a current and novel treatment method for COPD-emphysema diagnosed patients with or without psychiatric comorbidity has a positive impact on anxiety and depressive symptoms.

## 1. Introduction

Chronic obstructive pulmonary disease (COPD) is a frequently observed preventable and treatable disease that is characterized by permanent airway obstruction and respiratory symptoms mostly due to significant exposure to toxic particles and gases, airway, and/or alveolar anomalies [1]. The two primary components of COPD are chronic bronchitis and emphysema [2]. Estimated COPD prevalence is 11.2% [3], while estimated emphysema prevalence is 1.8% [4].

In addition to being a chronic physical disease, COPD also has mental, emotional, cognitive, social, and economic impacts on the individual resulting in many different issues and conflicts [5, 6]. In addition to making its primary impact on the lungs, it is also known as a multicomponent disease most likely due to chronic systemic inflammation and comorbidities may be observed at every stage of the disease [7]. Mental disorders, especially anxiety and depression, are among the most frequently encountered comorbidities [6, 8–10]. While anxiety prevalence in COPD varies between 2 and 96%, depression prevalence was determined to vary between 8 and 80% [8–11]. Studies indicate that this accompaniment yields significant results such as physical limitations, decrease in the quality of life, and increases in exacerbations and hospitalizations with impacts on treatment success, mortality, and morbidity [8–13]. Briefly, COPD affects peoples physically, emotionally, and socially. Anxiety and depression may occur due to the effects of COPD on brain functions, quality of life, and respiratory functions. Also symptoms of the disease like dyspnea can cause mental problems. While anxiety can be diagnosed earlier, depression is often noticeable late. Mental diseases adversely affect the prognosis in COPD and they are not adequately evaluated in patients unfortunately [14].

Emphysema is an advancing disease characterized by irrecoverable damages in the alveolar tissue. It manifests itself via reduction in pulmonary elastic recoil, hyperinflation, and increased air trapping. As a result, the lung can no longer flex in the rigid rib cage and fails to function [15, 16]. Treatment approaches applied for COPD are quitting smoking, bronchodilator, anti-inflammatory, mucolytic agents, pulmonary rehabilitation, nutrition support, vaccination, and long-term oxygen treatment, however, with limited effects since the real issue in emphysema is hyperinflation due to damages in the elastic tissue [15, 17]. This has led to the search for new kinds of treatment and lung-volume-reduction (LVR) treatments have come up especially for late stage emphysema patients [15, 17]. LVR treatments were first applied in 1954 but failed to find sufficient use in clinical applications; however, their effectiveness was rediscovered during the 1990s [18]. The coil (Coil, PneumRx Inc. Mountain View, California, USA) as one of the bronchoscopic LVR methods involves the use of nitinol wires shaped into a coil shriveling the lung parenchyma; they are placed in thereby reducing the excessive ventilation and hyperinflation in the lungs [15, 18]. It has been reported that decrease in hyperinflation reduces dyspnea, while increasing exercise capacity [19].

In the light of all these findings, we are of the opinions that coil treatment used especially on emphysema patients reduces dyspnea and disease severity and hence it may also reduce anxiety and depressive symptoms especially when the complex relationship between mental disorders and dyspnea is taken into consideration. The purpose of the present study was to examine the impact of coil treatment on accompanying anxiety and depressive symptoms in late stage COPD-emphysema diagnosed patients. There is only one study [20] in literature that examines psychiatric symptoms in patients treated with coil. In our study, the severity of anxiety and depressive symptoms was measured by using a different scale. Also we analyzed anxiety and depressive symptoms before and after the coil treatment among patients with and without psychiatric illness, respectively.

## 2. Materials and Methods

### 2.1. Participants

The study was designed as a prospective follow-up study (pretest and posttest design). Following the ethics council approval, between June and December 2019, a total of 27 patients followed up by University Faculty of Medicine Pulmonary Diseases polyclinic were included in the study. The inclusion criteria were severe and very severe airflow obstruction (i.e., Global Initiative for Chronic Obstructive Lung Disease (GOLD) [1] stage 3, stage 4, FEV_1_ 20–45%), patients who are highly symptomatic (grades C and D; Cognitive Ability test scores ≥ 10, modified British Medical Research Council scores ≥ 2; hyperinflation, i.e., residual volume (RV) ≥ 175% or RV/total lung capacity ≥ 0.58; a reduced 6-minute walk distance of 100–500 m) [16], and patients who have agreed to take part in the study. The exclusion criteria were postbronchodilator change in FEV_1_ ≥ 20%, frequent COPD exacerbation episodes (>2 hospitalizations per year), pulmonary artery pressure > 50 mmHg, giant bullae > 1/3 of a single lung volume, bronchiectasis, lung cancer, or use of an oral anticoagulant [16]. Also during the process, two patients were excluded when they died with COPD exacerbation, one patient was excluded upon rejecting to attend the control, and three patients were excluded when they could not be contacted, thus completing the study with a total of 21 patients. Seven patients had a psychiatric diagnosis and were receiving treatment. No new psychiatric medications or psychotherapy was performed during the measurement process. Also the data of these patients are further analyzed. All patients had completed their pulmonary rehabilitation programs prior to the procedure. A survey form including sociodemographic data as well as data that may be related to the disease (COPD) and anxiety/depression prepared by the researchers was applied to the patients prior to the study. Beck anxiety inventories (BAI) and beck depression inventory (BDI-I) were applied face to face by specialist doctors on the patients prior to the coil treatment and during the one month controls after the procedure. Written consents were taken from all participants prior to the study.

#### 2.1.1. Ethical Statement

This study was carried out in accordance with the Helsinki Declaration and approved by the University Ethics Council with the decree numbered 10 and dated 21.05.2019.

### 2.2. Procedure

#### 2.2.1. Coil Application

The procedure is applied under general anesthesia in the accompaniment of fluoroscopy. At first, only one lobe is treated and the other targeted lobe in the opposite lung is treated 4–8 weeks later. First, the airway in the selected segment is determined bronchoscopically and its length is measured with the help of a guide wire. The coil is placed inside the targeted segment using a carrier catheter which takes on the original shape. The airway shrinks as the coil pulls the lobe and the lung collapses and shrinks. The targeted lobe is systematically treated with an average of 10–14 coils [21].

### 2.3. Tools of Measurement

#### 2.3.1. Beck Anxiety Inventory (BAI)

A Likert-type self-report inventory applied for determining the prevalence and intensity of the anxiety symptoms experienced by the individual: the BAI scale evaluates subjective anxiety and somatic symptoms like dizzy, difficulty in breathing, lightheaded, flushing, and heart pounding. The highest score that can be obtained from the inventory is 63 and covers 21 symptoms. The BAI showed a high internal consistency (alpha: 0.93) and the Turkish validity and reliability study was carried out by Ulusoy et al. [22].

#### 2.3.2. Beck Depression Inventory (BDI-I)

A Likert-type self-report inventory for measuring the level and severity of depressive symptoms: the BDI-I (original version) scale evaluates somatic, emotional, cognitive, vegetative, and impulsive symptoms of depression. The scale comprised of four levels (between 0 and 3) and 21 items provides information on the severity of depressive symptoms through its total score. The validity and reliability study for the Turkish form was carried out by Hisli [23] and the Cronbach's alpha value of the BDI-I scale was found to be 0.80.

### 2.4. Statistical Analyses

Statistical Package for the Social Sciences (SPSS v21, Chicago, Illinois, USA) software was used for the statistical analyses of the data obtained in our study. As a result of the power analysis conducted, it was calculated that when at least 17 patients were included in the study, a 95% confidence interval and 80% power could be obtained. Frequency (*n*), percentage (%), mean, and standard deviation (SS) were used as descriptive statistics. The chi square (*χ*^2^) test was used for examining the categorical variables. Compliance of the variables to normal distribution was examined via the Shapiro–Wilk test for normality and graphical methods. The Wilcoxon signed-rank test was used for dependent group comparisons and Mann–Whitney *U* test used for independent group comparisons when parametric test assumptions could not be met. Spearman correlation coefficient was used for examining the relationship between the variables. Statistical significance was considered as *p* < 0.05 for all tests.

## 3. Results

The study was completed by collecting the data for the 21 patients subject to coil treatment with emphysema diagnosis. All patients were male with a mean age of 66.5 ± 5.5 (57–76). With regard to education status, 57.1% (*n* = 12) were primary school graduates, whereas 19.1% (*n* = 4) were high school and 23.8% (*n* = 5) were university graduates. All patients were married.

It was determined that when various parameters related to the disease were examined with regard to COPD, 4.8% (*n* = 1) start again smoking after coil treatment, whereas 95.2% (*n* = 20) were ex-smoker. Mean smoking rate was 58.4 ± 25.5 (30–100) pack-year. The duration of time that passed after COPD diagnosis was determined as 10.6 ± 7.3 (1–28) years. The psychiatric disease and treatment histories of the patients were recorded. Accordingly, while 33.3% (*n* = 7) of the patients were determined to be subject to treatment for a psychiatric disorder, a diagnosis could not be determined in 66.7% (*n* = 14). The diagnoses were as bipolar disorder in one patient, anxiety disorder in three patients, and depressive disorder in three patients. While one of the patients was using a mood stabilizer, others were subject to antidepressant treatment. Sociodemographic and health-related variables are shown in Table 1.

A relationship could not be determined between age, smoking pack-year and COPD duration (year), and BAI and BDI-I scores prior to the coil procedure (*p* > 0.05). Scores were compared with the same data following the coil procedure as a result of which a statistically significant relationship could not be determined (*p* > 0.05) (Table 2).

BAI and BDI-I scores comparisons of patients with and without psychiatric diagnosis were analyzed before and after the coil treatment and were found similar (Table 3).

BAI scores were compared before and after coil which were determined, respectively, as 12.1 ± 6.3 (4–26) and 11.2 ± 9.3 (0–28) in the patients without psychiatric diagnosis (*W*=−0.455; *p*=0.649). BDI-I scores before and after coil were determined, respectively, as 13.5 ± 10.4 (1–31) and 8.8 ± 10.6 (0–34) with a statistically significant difference (*W*=−2.451; *p*=0.014). While anxiety symptoms of the patients in our study decreased slightly after the procedure, the difference was not statistically significant; however, it was observed that depressive symptoms decreased at a statistically significant level. In the patients with psychiatric diagnosis, BAI scores were also compared before and after coil which were determined, respectively, as 15.0 ± 7.8 (3–23) and 9.2 ± 4.9 (2–17) (*W*=−2.201; *p*=0.028). BDI-I scores were determined, respectively, as 15.1 ± 11.1 (4–31) and 7.1 ± 4.9 (2–16) (*W*=−1.782; *p*=0.075). BAI scores of patients with psychiatric diagnosis were significantly decreased after the coil treatment. Also BDI-I scores decreased but were not statistically significant (Table 4).

## 4. Discussion

It was determined in our study that coil treatment on patients diagnosed with late stage COPD-emphysema has a positive impact on the accompanying anxiety and depressive symptoms and that the impact on depressive symptoms is more significant. Although anxiety and depressive symptoms decreased in cases with comorbid psychiatric diagnosis, the decrease in anxiety was found significant.

We think that the effects of comorbid psychiatric symptoms or diagnosis on mortality and morbidity in COPD may affect the adaptive skills to the treatments like coil as a promising and expensive treatment. There is only one study [20] in the literature on coil therapy and its effects on psychiatric symptoms. This study aims to present the authors' experience with endobronchial coils. They looked at anxiety and depression scores as well as many dependent variables in the study. In our study, comprehensive and valid scales were used to measure anxiety and depressive symptoms. Additionally, the hypothesis of our study is that anxiety and depressive symptoms of COPD are affected by coil therapy. So we think that our study is important in terms of providing us to look at coil treatment from a different perspective, as well as drawing attention to psychiatric conditions that are frequently accompanied and can be unheeded despite its importance.

Even though the mechanism for the association of COPD and anxiety and depression which are among the primary mental comorbidities cannot be explained clearly, it is considered to develop secondary to systemic inflation, dyspnea, smoking, hypoxia, oxidative stress, airway blockage, physical limitation, loss of control in the living area and the disease, reduced self-esteem, and self-confidence [5, 10, 14, 24]. Dyspnea is the most frequently observed symptom in COPD causing significant problems and loss of functions as well as limitations. Dyspnea is observed in 82% of the patients regardless of stage [25]. While patients define the dyspnea-related symptoms experienced during periods of exacerbation as anxiety, they evaluate the increased anxiety symptoms as an indication that the disease will exacerbate [10].

A relationship could not be determined in our study between anxiety, depression scores prior to coil treatment, and smoking pack-year. The fact that all patients have a history of smoking does not enable us to evaluate the risk. Apart from smoking, a relationship could not be determined between anxiety and depression scores prior to the coil procedure between the disease durations which might be an indirect indication of symptoms such as physical limitation, function loss, and dyspnea due to the advancing nature of COPD. This may be explained by the fact that about one-third of the patients were on psychotropic medications with mental disorder diagnosis at the time they were included in the study.

Even though the decrease in the anxiety scale scores during evaluations before and after the procedure was not reflected in the statistics, a statistically significant decrease was observed in depression scores. The positive impact of the coil procedure on the lungs is shrinking the damaged emphysematous airways, thus reducing the lung volume leading to reduced hyperinflation and hence decreased dyspnea, decreased fast and surface respiration, reduction in airway resistance, and provision of air flow in healthy areas [26, 27]. A decrease in both anxiety and depressive symptoms can be expected when the impacts of the procedure are considered on dyspnea and related factors thought to be factors of COPD and mental comorbidity. Even though the BAI and BDI-I used in our study are among inventories suggested for use in this patient group [28], the fact that the somatic symptoms (tachycardia, stomach discomfort, labored breathing, sense of suffocation, perspiration, trembling, dizziness, weakness, etc.) overlapping with the COPD symptoms are questioned by scoring in especially the BAI makes it difficult to distinguish. This similarity may have affected anxiety-related results. The impacts of the coil procedure and its results on dyspnea may have indirectly reduced depressive symptoms since they may lead to an increase in the sense of hope and functionality of the patient and decrease in pessimism and social isolation in addition to improved quality of life. Besides the biological effects of the procedure, we think that the psychosocial effects may have reduced depressive symptoms to a greater extent.

The results of the limited number of studies on the use of antidepressants and anxiolytic agents in the treatment of the anxiety and depressive disorders accompanying COPD are observed to vary [29]. It is observed that the use of benzodiazepine is frequently preferred, that the compliance with medication of the patients is generally low, and that the impacts of advanced age along with the impacts of other accompanying physical comorbidities are frequently observed and there are also conflicting data on the use of antidepressant treatments [29]. In our study, when the data of patients with psychiatric comorbid diagnoses were also evaluated, they were currently using antidepressant and anxiolytic therapies when they were included in the study. After coil treatment, both anxiety and depressive symptoms decreased and this reduction was statistically significant for anxiety symptoms. The complex and intertwined perceptions of patients about COPD symptoms and anxiety may have influenced this result. COPD symptoms were sometimes seen as anxiety, and anxiety symptoms were sometimes seen as a symptom of disease or exacerbation, and this may lead to anticipatory anxiety especially in psychiatric comorbid patients. Coil as a promising treatment may have had a positive effect on anticipatory anxiety and altered cognitive beliefs of patients with comorbid psychiatric diagnosis. Bostancı et al. found significant decrease in both anxiety and depression scores [20]. In this study, second measurements recorded six months later the coil treatment and it was not stated whether psychiatric comorbid diagnoses are excluded or not. Also pretreatment anxiety and depression scores were lower than our study. These may have affected the difference between the results. Additionally, the short follow-up period of our study may have caused the long-term effects not to be seen.

The fact that coil treatment is expensive requires further education on top of specialist skills; that can be applied only in a few centers in our country and only on a small group of patients that meet certain criteria reflected in our study as a low sample size which makes up the most important limitation of our study. Small sample size also reduces the statistical strength of the analyses. Secondly, the short follow-up period also restricts us from measuring whether treatment has a chronic effect on psychiatric symptoms.

In conclusion, coil treatment as a novel and current form of treatment for patients diagnosed with late stage COPD-emphysema has a positive impact on anxiety which is one of the prevalent comorbidities in this patient group, while reducing depressive symptoms at a statistically significant level. Moreover, coil treatment reduces anxiety and depressive symptoms in patients with comorbid psychiatric diagnosis too and this time its effect on anxiety is more significant. It is suggested to support the results by way of randomized controlled studies with larger sample sizes.

## Figures and Tables

**Table 1:** Sociodemographic and health-related variables.

		*n*	%
Gender	Male	21	100.0
Marital status	Married	21	100.0
Education	Primary school	12	57.1
	High school	4	19.1
	University	5	23.8
Smoking	Ex-smoker	20	95.2
	Start again smoking after coil treatment	1	4.8
The psychiatric disease and treatment histories	No	14	66.7
	Yes	7	33.3

		Mean ± SD	Min–max

Age		66.5 ± 5.5	57–76
Mean smoking rate		58.4 ± 25.5	30–100
The duration of time that passed after COPD diagnosis (year)		10.6 ± 7.3	1–28

*n*, number; %, percentage; SD, standard deviation; min, minimum; max, maximum; COPD, chronic obstructive pulmonary disease.

**Table 2:** The relationship between age, smoking pack-year, and COPD duration, beck anxiety, and beck depression scores before and after coil treatment.

	Test	BAI (pre)	BDI-I (pre)	BAI (post)	BDI-I (post)
Age	Correlation	0.109	0.393	0.061	0.287
	*p*	0.637	0.078	0.793	0.208
	*n*	21	21	21	21

Smoking pack-year	Correlation	0.150	0.165	−0.060	−0.057
	*p*	0.517	0.475	0.796	0.805
	*n*	21	21	21	21

COPD duration (year)	Correlation	0.291	−0.073	0.148	−0.156
	*p*	0.200	0.753	0.522	0.500
	*n*	21	21	21	21

BAI (pre), precoil beck anxiety inventory; BDI-I (pre), precoil beck depression inventory; BAI (post), postcoil beck anxiety inventory; BDI-I (post), postcoil beck depression inventory.

**Table 3:** Comparisons of beck anxiety and beck depression scores before and after coil treatment of patients with and without psychiatric diagnosis.

		Mean ± SD	*U*	*z*	*p*
BAI (pre)	Patients without PD	12.1 ± 6.3	39.500	−0.711	0.488
	Patients with PD	15.0 ± 7.8			

BAI (post)	Patients without PD	11.2 ± 9.3	49.000	0.000	1.000
	Patients with PD	9.2 ± 4.9			

BDI-I (pre)	Patients without PD	13.5 ± 10.4	42.500	−0.486	0.636
	Patients with PD	15.1 ± 11.1			

BDI-I (post)	Patients without PD	8.8 ± 10.6	43.500	−0.412	0.689
	Patients with PD	7.1 ± 4.9			

BAI (pre), precoil beck anxiety inventory; BDI-I (pre), precoil beck depression inventory; BAI (post), postcoil beck anxiety inventory; BDI-I (post), postcoil beck depression inventory; PD, psychiatric diagnosis; SD, standard deviation; *U*, Mann–Whitney *U* test; *z*, Mann–Whitney *U* test statistic.

**Table 4:** The comparison of beck anxiety and beck depression scores before and after coil treatment for both patients with and without psychiatric diagnosis.

	Scale	Precoil		Postcoil		*z*	*p*
		Mean ± SD	Min–max	Mean ± SD	Min–max		
Patients without PD	BAI	12.1 ± 6.3	4–26	11.2 ± 9.3	0–28	−0.455	0.649
	BDI-I	13.5 ± 10.4	1–31	8.8 ± 10.6	0–34	−2.451	**0.014**

Patients with PD	BAI	15.0 ± 7.8	3–23	9.2 ± 4.9	2–17	−2.201	**0.028**
	BDI-I	15.1 ± 11.1	4–31	7.1 ± 4.9	2–16	−1.782	0.075

BAI, beck anxiety inventory; BDI-I, beck depression inventory; SD, standard deviation; min, minimum; max, maximum; PD, psychiatric diagnosis; *z*, Wilcoxon test statistic.

## Data Availability

The sociodemographic and scales related data used to support the findings of this study are included within the article and are available from the corresponding author upon request.
